# Recent Advances in the Distribution, Chemical Composition, Health Benefits, and Application of the Fruit of *Siraitia grosvenorii*

**DOI:** 10.3390/foods13142278

**Published:** 2024-07-19

**Authors:** Qihan Guo, Minke Shi, Sarengaowa, Zhewen Xiao, Ying Xiao, Ke Feng

**Affiliations:** 1 Medical Science Division, Macau University of Science and Technology, Macao 999078, Chinaminkeshi8@gmail.com (M.S.); 13681245239@163.com (Z.X.); yxiao@must.edu.mo (Y.X.); 2 College of Life Science, Zhuhai College of Science and Technology, Zhuhai 519041, China

**Keywords:** *Siraitia grosvenorii*, chemical composition, health benefits, application

## Abstract

The fruits of *Siraitia grosvenorii* (*S. grosvenorii*) have attracted a lot of scientific interest as part of the current healthy diet. *S. grosvenorii* has diverse health-promoting effects, including antioxidant, anti-inflammatory, antimicrobial, respiratory modulation, metabolic modulation, antitumor, and neuroprotective effects, as well as gastrointestinal function modulation. As a plant resource, *S. grosvenorii* has broad application prospects, which promotes the development of the horticultural industry. Moreover, Mogroside has attracted much attention as an important active ingredient of *S. grosvenorii*. This review provides an in-depth exploration of the distribution, chemical composition, health benefits, and application of *S. grosvenorii*, particularly Mogroside. This comprehensive exploration highlights the important therapeutic potential of *S. grosvenorii*, prompting further research into its applications. As value-added functional ingredients, *S. grosvenorii* and its constituents have significant potential for disease prevention and are widely used in the development of food and health supplements.

## 1. Introduction

*Siraitia grosvenorii* (*S. grosvenorii*), commonly known as Monk fruit, belongs to the Cucurbitaceae Juss family and is the fruit of *S. grosvenorii* (Swingle) C. Jeffrey ex A. M. Lu & Zhi Y. Zhang, a unique variety of the genus Siraitia Merr found in China [1,2]. Its history of cultivation and use reaches back hundreds of years in the Guilin region of China. The association of *S. grosvenorii* with Guilin is also an agro-product geographical indication (APGI) [3,4]. Fresh *S. grosvenorii* fruit’s texture is crisp, and its sweet taste means it is widely consumed in China and Japan. As a natural product, *S. grosvenorii* has a wide range of food application and medicine prospects [5]. The extract of *S. grosvenorii* is usually used as a natural sweetener instead of sucrose because it is rich in Mogroside, low in calories, and very sweet, properties that mean it can improve the taste of food [6]. It is widely used in a variety of foods and beverages, especially health foods and weight loss products. *S. grosvenorii* also has antioxidant and antibacterial effects, which can extend the shelf life of food [7,8]. *S. grosvenorii* fruit extracts have been granted GRAS (generally recognized as safe) status by the U.S. Food and Drug Administration (FDA) [9]. They have also been approved for use as Intense Sweeteners by Food Standards Australia New Zealand (FSANZ) [10]. Momordica glycosides, commonly known as Mogrosides, are natural sweeteners found in the fruit of the *Siraitia grosvenorii* plant. They comprise triterpenoid saponins and their derivatives. These compounds have captured considerable attention in recent years because they have intense sweetness without the addition of calories, making them a popular choice of sugar substitute in various food and beverage products. Within the category of Mogrosides, the most abundant, Mogroside V, is up to 425 times sweeter than sucrose. Meanwhile, Siamenoside I is the sweetest of the Mogrosides, at 563 times sweeter than 5% sucrose at a concentration of one part per million [11,12]. Research on Mogrosides has highlighted their potential health benefits, including antioxidant and anti-inflammatory properties, as well as their role in managing blood sugar levels, making them appealing for pharmaceutical and functional food applications [13]. In addition, *S. grosvenorii* has certain medicinal value and can be used to make dietary supplements or medicinal ingredients to help regulate blood sugar, lower blood pressure, and enhance immunity and other effects. It is also often used in traditional medicinal diets. Modern studies have confirmed that *S. grosvenorii* engages in various physiological activities, including antioxidant, hypoglycemic, lipid-regulating, hepatoprotective, and antitumor activities, which are closely related to its chemical components.

## 2. Botanical Characterization and Distribution

**Figure 1:**
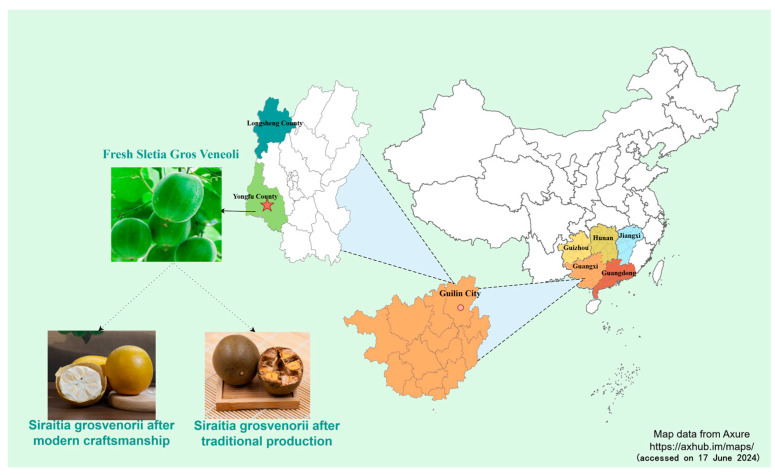
*S. grosvenorii* planting distribution map. https://axhub.im/maps/ (accessed on 17 June 2024).

*S. grosvenorii* is a perennial climbing herb with leaf blades measuring 12–23 cm in length and 5–17 cm in width. The petiole ranges from 3 to 10 cm. Its leaves are typically ovate–cordate, triangular–ovate, or broadly ovate–cordate in shape. The fruit is spherical or oblong, 6–11 cm long, and 4–8 cm in diameter. The surface of the fruit is brown, yellow–brown, or green–brown in color, with yellow–brown hairs and mixed black glandular scales on the surface of the fruit, which fall off at maturity, and yellow–brown or green–brown tomentum, which is very pale. The pericarp is thin and brittle when dry. Its seeds are numerous, yellowish, suborbicular or broadly ovoid, flattened, 15–18 mm long, and 10–12 mm wide. Plants flower from May to July and fruit from July to September [14,15]. This plant has specific environmental requirements, typically thriving at the bases of hillsides and in riverside wetlands. It grows as a shrub at altitudes between 400 and 1400 m. *S. grosvenorii* is distributed in Guangxi, Guizhou, southern Hunan, Guangdong, and Jiangxi in China, with primary production centered in Yongfu County and Longsheng County, Guilin City, Guangxi, China [1,14,16,17] (Figure 1). The varietal classification of *S. grosvenorii* is mainly based on the shape of the fruit. According to the *S. grosvenorii* fruit shape and origin, the plants can be categorized as Long Beach Fruit, Lajiang Fruit, Winter Melon Fruit, Green Fruit, etc. In a survey taken in the 1980s, the Guangxi Institute of Botany discovered 13 wild varieties of *S. grosvenorii*. Since then, the number of wild germplasm resources of *S. grosvenorii* has decreased continually, and some varieties of wild germplasm resources have become extinct. Researchers have used aerospace technology for aeronautical breeding to improve the variety of *S. grosvenorii*. At present, the *S. grosvenorii* that can be purchased in the market is generally Longjiang NO.1, Long Beach Fruit, or Lajiang Fruit [5,18]. *S. grosvenorii* was selected as a Chinese agricultural product with a geographical indication in 2018 under the name “Guilin Luo Han Gu”. As of 2022, the planting output of *S. grosvenorii* in Guilin City had reached 203,800 tons. As one of the main producing areas of *S. grosvenorii*, Yongfu County has an annual processing capacity of more than 5 billion *S. grosvenorii*, with an annual output value of CNY 7 billion. More than 70% of *S. grosvenorii* in the world is processed and sold in Yongfu County [3].

## 3. Chemical Composition

**Figure 2:**
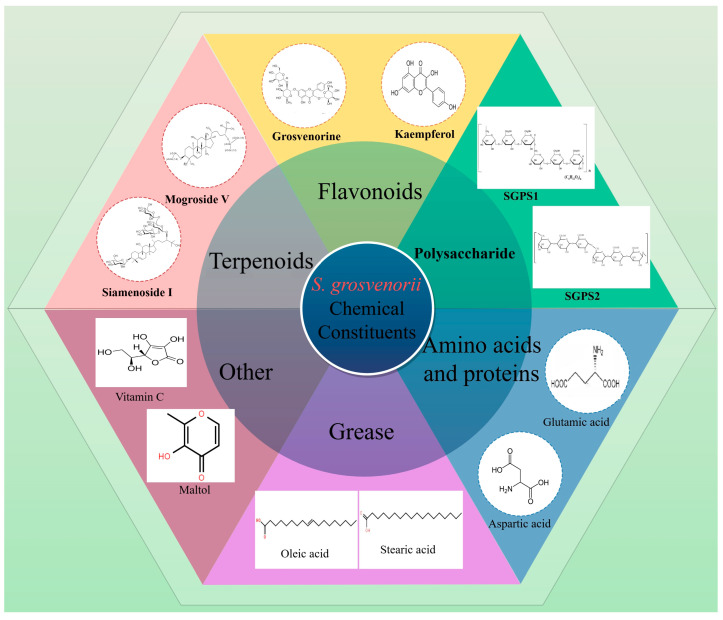
Chemical constituents in *S. grosvenorii*.

A plethora of chemicals can be identified in *S. grosvenorii*, including terpenoids, flavonoids, polysaccharides, amino acids and proteins, grease, and others [19] (Figure 2). Among these, the triterpene saponins stand out for having notable antioxidant and anti-inflammatory properties in *S. grosvenorii*, garnering significant research attention [20].

### 3.1. Terpenoids

**Figure 3:**
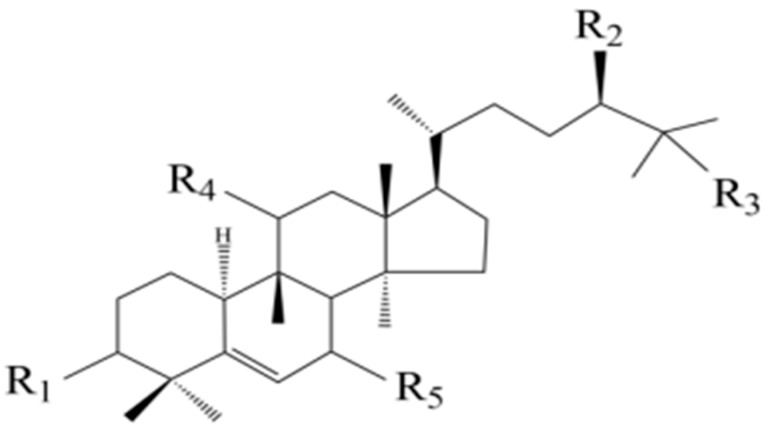
Mogroside mother nucleus structure.

**Figure 4:**
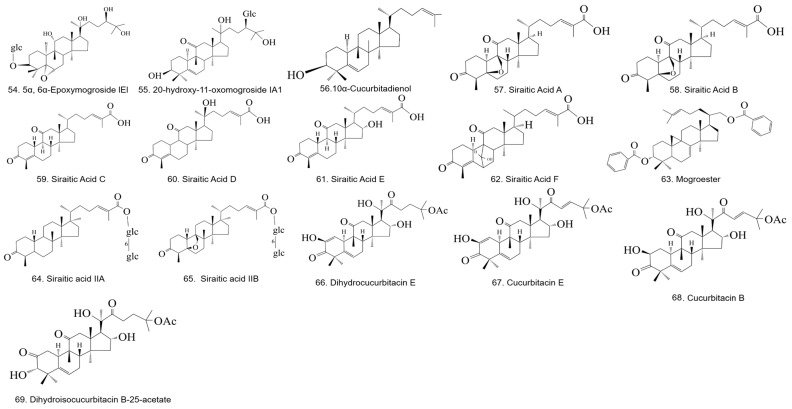
Structures of triterpenoids from *S. grosvenorii*.

Since the isolation of triterpene glycosides from *S. grosvenorii* by researchers in 1975 [21], the chemical composition of *S. grosvenorii* has been studied in detail, leading to the isolation and identification of a variety of *S. grosvenorii* triterpene saponins, which are the main chemical and active ingredients and mostly have a sweet flavor. The triterpene glycosides extracted from *S. grosvenorii* are also known as Mogrosides [20]. Mogrosides form a unique class of Cucurbitane-type tetracyclic triterpenoid substances in *S. grosvenorii*. Cucurbitane-type tetracyclic triterpenoids have a variety of pharmacological activities, such as antitumor, anti-inflammatory, hypoglycemic, and antioxidant effects, and are also the most studied class of chemical components in *S. grosvenorii*. Moreover, Mogrosides are the main source of sweetness in *S. grosvenorii*, and the non-sugar sweet components included are mainly triterpene glycosides: Mogroside IV, Mogroside V, and Siamenoside I [12]. More detail is given in Figure 3 and Figure 4 and Table 1.

**Table 1:** Structures of triterpenoids from *S. grosvenorii* ^1,2^.

No.	Compound Name	R1	R2	R3	R4	R5	Pharmacological Activity	Ref.
1	Mogrol	β-OH	OH	OH	α-OH, H	H2	Neuroprotective; inhibition of the production of inflammatory mediators; inhibition of adipocyte differentiation; inhibitory effects against the Epstein–Barr virus early antigen	[22,23,24,25,26]
2	Mogroside IA (Mogroside IA1)	β-OH	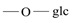	OH	H2	H2	Inhibitory effects against the Epstein–Barr virus early antigen	[23,24,27]
3	Mogroside IE (Mogroside IE1)	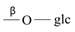	OH	OH	α-OH, H	H2	Inhibitory effects against the Epstein–Barr virus early antigen	[23,24,28]
4	Mogroside IIA1	β-OH	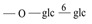	OH	α-OH, H	H2	Inhibitory effects against the Epstein–Barr virus early antigen	[27,29]
5	Mogroside IIA2	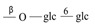	OH	OH	α-OH, H	H2	-	[30]
6	Mogroside IIE	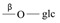	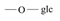	OH	α-OH, H	H2	Inhibitory effects against the Epstein–Barr virus early antigen	[23,24,28,31,32,33]
7	Mogroside IIB	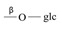	OH	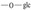	α-OH, H	H2	Inhibitory effects against the Epstein–Barr virus early antigen	[29]
8	Mogroside III	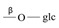	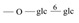	OH	α-OH, H	H2	Inhibitory effects against the Epstein–Barr virus early antigen	[24,28,30,31,32,33]
9	Mogroside IIIA1	β-OH	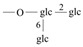	OH	α-OH, H	H3	-	[27,30]
10	Mogroside IIIA2	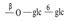	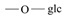	OH	α-OH, H	H2	Inhibitory effects against the Epstein–Barr virus early antigen	[29]
11	Mogroside IIIE	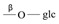	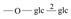	OH	α-OH, H	H2	-	[23,28,30]
12	Mogroside IVA	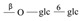	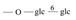	OH	α-OH, H	H2	Inhibitory effects against the Epstein–Barr virus early antigen	[24,30,32]
13	Mogroside IVE	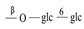	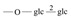	OH	α-OH, H	H2	Inhibitory effects against the Epstein–Barr virus early antigen	[23,24,30]
14	Mogroside V	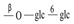	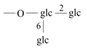	OH	α-OH, H	H2	Inhibitory effects against the Epstein–Barr virus early antigen	[23,24,27,28,30,31,32,33,34,35]
15	Mogroside VA1	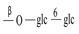	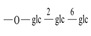	OH	α-OH, H	H2	-	[34]
16	Mogroside VIA	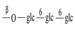	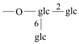	OH	α-OH, H	H2	-	[30,36]
17	Mogroside VIB	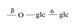	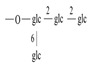	OH	α-OH, H	H2	-	[36]
18	Siamenoside I	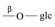	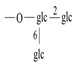	OH	α-OH, H	H2	Inhibitory effects against the Epstein–Barr virus early antigen	[24,28,30]
19	Neomogroside	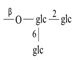	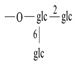	OH	α-OH, H	H2	-	[28]
20	Grosmomoside I	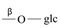	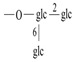	OH	α-OH, H	H2	-	[31]
21	Isomogroside V	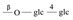	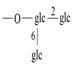	OH	α-OH, H	H2	-	[30,35]
22	Isomogroside IVa	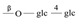	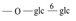	OH	α-OH, H	H2	-	[30]
23	Isomogroside IVe	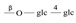	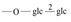	OH	α-OH, H	H2	-	[30]
24	11-Oxomogrol	β-OH	OH	OH	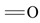	H2	Inhibitory effects against the Epstein–Barr virus early antigen	[24]
25	11-Oxomogroside IA1 (11-Oxomogroside A1)	β-OH	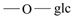	OH	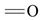	H2	Inhibitory effects against the Epstein–Barr virus early antigen	[24,28,32,33]
26	11-Oxomogroside IE1	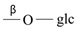	OH	OH	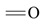	H2	Inhibitory effects against the Epstein–Barr virus early antigen	[24]
27	11-Oxomogroside IIA1	β-OH	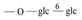	OH	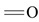	H2	Inhibitory effects against the Epstein–Barr virus early antigen	[29]
28	11-Oxomogroside IIE	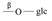	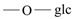	OH	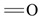	H2	-	[32,33]
29	11-Oxomogroside III	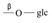	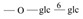	OH	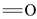	H2	-	[33]
30	11-Oxomogroside III A1	β-OH	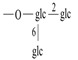	OH	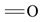	H2	-	[37]
31	7β-Methoxy-mogroside V	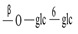	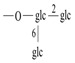	OH	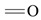	H,OMe	-	[37]
32	11-Oxomogroside IIIE	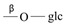	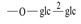	OH	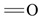	H2	-	[36]
33	11-Oxomogroside IVA	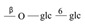	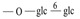	OH	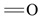	H2	Inhibitory effects against the Epstein–Barr virus early antigen	[29]
34	11-Oxomogroside IV	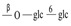	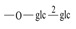	OH	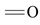	H2	-	[33,36]
35	11-Oxomogroside V	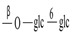	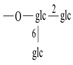	OH	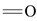	H2	Inhibitory effects against the Epstein–Barr virus early antigen	[24,28,30]
36	11-Oxoisomogroside V	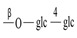	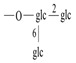	OH	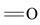	H2	-	[36]
37	11-Oxomogroside VI	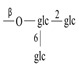	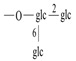	OH	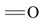	H2	-	[30]
38	11-Oxosiamenoside I	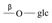	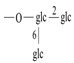	OH	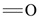	H2	-	[30]
39	7-Oxomogroside IIE	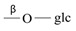	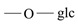	OH	α-OH, H	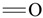	Inhibitory effects against the Epstein–Barr virus early antigen	[29]
40	7-Oxomogroside V	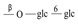	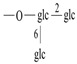	OH	α-OH, H	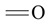	Inhibitory effects against the Epstein–Barr virus early antigen	[29]
41	7-Oxomogroside IIIE	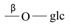	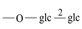	OH	α-OH, H	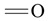	-	[36]
42	7-Oxomogroside IV	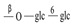	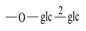	OH	α-OH, H	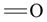	-	[36]
43	11-Deoxymogroside III (11-Dehydroxymogroside III)	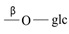	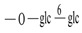	OH	H2	H2	Inhibitory effects against the Epstein–Barr virus early antigen	[29,33]
44	11-Epi-mogroside V	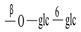	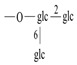	OH	β-OH,H	H2	-	[30]
45	25-Methoxy-11-oxomogrol	β-OH	OH	OMe	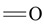	H2	-	[25]
46	25-Methoxymogrol	β-OH	OH	OMe	β-OH,H	H2	-	[25]
47	25-Dehydroxy-24-oxomogrol	β-OH	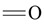	H	β-OH,H	H2	-	[25]
48	3-Hydroxy-25-dehydroxy-24-oxomogrol	α-OH	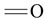	H	β-OH,H	H2	AMPK activators	[25]
49	3α-Hydroxymogrol	α-OH	OH	OH	α-OH,H	H2	AMPK activators	[25]
50	Bryogenin	β-OH	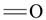	H	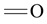	H2	-	[25]
51	11-Deoxymogroside V	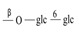	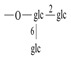	OH	H2	H2	-	[30,35]
52	11-Deoxyisomogroside V	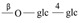	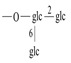	OH	H2	H2	-	[35]
53	11-Deoxymogroside VI	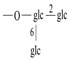	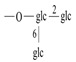	OH	H2	H2	-	[35]

^1^ Component parent nuclei in the table are all Figure 3 structures. ^2^ The figure shows the structures of triterpenoids from *S. grosvenorii*.

### 3.2. Flavonoids

Flavonoids are a class of polyphenolic compounds with flavonoid structures, which are widely found in plants. They are generally divided into flavonoids, isoflavones, flavonoids, flavanes, and flavonoid glycosides [38]. Flavonoids have a variety of biological activities, such as antioxidant, anti-inflammatory, and anti-cancer effects, among others [39,40,41,42,43]. Common flavonoids include quercetin, soy isoflavones, apigenin, and kaempferol [38,44]. The flavonoids in *S. grosvenorii* are mainly quercetin and kaempferol, as well as different glycoside derivatives with these two flavonoids as the mother nucleus [45]. *S. grosvenorii* contains very few flavonoids, and regarding the study of flavonoids in *S. grosvenorii*, only superficial studies have been carried out so far. The flavonoids found in *S. grosvenorii* with quercetin and kaempferol as the basic units are kaempferol-3-O-α-L-rhamnosyl-7-O-[β-D-glucosyl-(1-2)-α-L-rhamnoside] (Grosvenorine), kaempferol-3,7-α-L-di-rhamnopyranoside [46], kaempferol [47], and kaempferitrin [48]. Researchers have isolated the flavonoid glycoside quercetin from the fresh fruits of *S. grosvenorii* and determined a total flavonoid content of 5–10 mg in one fresh *S. grosvenorii* by RP-HPLC using quercetin and kaempferol as controls [49]. Studies have shown that the flavonoids of *S. grosvenorii* have antioxidant, antibacterial, and blood-glucose-lowering physiological activities. Its anti-oxidizing capacity can reach four times that of BHT (butylated hydroxytoluene) [50]. The flavonoid extract (which reached 4.00 mg/mL) showed bacteriostatic effects against *Escherichia coli*, *Staphylococcus aureus*, *Bacillus subtilis*, *Pseudomonas aeruginosa*, *Rhizopus*, and *Aspergillus* [51]. It significantly improved body weight in the treatment of streptozotocin (STZ)-induced type II diabetic rats [52]. The total flavonoids of *S. grosvenorii* could alleviate oxidative stress damage and inflammatory responses caused by chronic sleep deprivation, and it modulated the expression of antioxidant factors such as T-AOC and HO-l, as well as MDA and inflammatory factors and their related genes, in the sera and brains of chronic sleep-deprived mice [53]. In addition, the flavonoids in *S. grosvenorii* also have anti-aging [54], anti-atherosclerosis [55], and hepatoprotective [56] effects. 

### 3.3. Polysaccharides

Polysaccharides, produced by plant cell metabolism, are macro-structures of >10 monosaccharides linked by glycosidic bonds [57]. Plant polysaccharides boost immunity, regulate lipids, fight viruses, lower blood sugar, antioxidize, and have anti-inflammatory and antitumor properties [57,58,59]. Polysaccharides in *S. grosvenorii* are an important class of bioactive components and mainly include *S. grosvenorii* polysaccharides, glucosides, etc. These polysaccharide compounds not only give *S. grosvenorii* its unique taste and sweetness but may also have nutritional health and physiological regulatory effects [7,60,61,62,63,64,65,66]. Their presence in *S. grosvenorii* enriches its nutritional value, making it a popular natural sweetener and health food ingredient. So far, SGPS1, SGPS2, and SGP have been isolated from *S. grosvenorii*; further studies have found that the polysaccharide SGPS1 is an acidic heteropolysaccharide, composed of glucose, galactose, rhamnose, xylose, and arabinose. The molar ratio of each sugar residue is Rha:Ara:Xyl:Gal:Glc:GlcA = 1.00:2.30:1.40:9.70:39.53:2.46 [67]. SGPS2 is a polysaccharide composed of rhamnose and glucuronic acid with a relative molecular weight of 650,000, consisting of (1→2,4) Rha and (1→4) Rha as the backbone, (1→2) Rha as the side chain, and (1→3) Rha fragments, which are also terminal groups, and GlcA exists in the molecule as terminal GlcA and 2-position substituted GlcA. SGPS2 for each sugar residue has a Molar Rha: GlcA = 8.24:0.99 [68]. SGP is composed of α-L arabinose, α-D-mannose, α-D-glucose, α-D-galactose, glucuronic acid, and galacturonic acid in the proportions 1:1.92:3.98:7.63:1.85:7.34 [7]. The backbone is made up of galactose, which is linked by an α-(1,4)-glycosidic bond. Branched chains include the α-1,6 linked glucose branch, α-1,6 linked mannose branch, α-1,3 linked galactose branch, and arabinose branch (α-L-Ara (1→)) [69]. *S. grosvenorii* polysaccharides have antioxidant and immunomodulatory effects. *S. grosvenorii* polysaccharides can downregulate histamine in mast cells and inhibit nose-grabbing behavior in mice stimulated by it [70]. Polysaccharides can antagonize the immunosuppressive effects caused by cyclophosphamide [71]. The polysaccharides in *S. grosvenorii* can promote the proliferation of RAW264.7 cells, can significantly promote the cellular secretion of NO, IL-6, and TNF-α, and increase cellular phagocytosis, with significant immunomodulatory activity [72]. The SGP in *S. grosvenorii* can affect the immune function by regulating the level of free radicals [73].

### 3.4. Amino Acids and Proteins

*S. grosvenorii* is a high-quality source of plant protein that helps maintain the normal function and health of body tissues. Researchers measured the protein content of dried monk fruit at 8.70%~13.35% and also determined the type and content of amino acids in the hydrolysate of dried *S. grosvenorii*, proving that the hydrolysate of dried *S. grosvenorii* contained 17 kinds of amino acids (except tryptophan), including 8 kinds of essential amino acids, with the highest contents of aspartic acid and glutamic acid [74]. Among them also were threonine, valine, leucine, isoleucine, phenylalanine, lysine, and methionine, the contents of which accounted for 31% of the total amino acids in *S. grosvenorii* [75]. Therefore, *S. grosvenorii* is not only favored for its unique taste and sweetness but is also considered a nutritious food for improved health due to its rich amino acid and protein contents [74,76].

### 3.5. Grease

The grease of *S. grosvenorii* is mainly derived from its seeds. The seeds of *S. grosvenorii* contain 27~33% grease [77]. It has been found that the grease of *S. grosvenorii* seeds contains several fatty aldehydes, such as farnesol, glutaraldehyde, hexanal, nonanal, palmitic acid, and decanal, of which farnesol is the major chemical constituent, accounting for 52.4% [77,78,79]. Farnesol is also known as 3,7,11-trimethyl-2,6,10-dodecatrien-1-ol. It is a colorless to slightly yellow oily liquid, usually used as a fragrance to make perfumes smell stronger or as a deodorant [80]. It is also used as a solvent and surfactant. In addition, farnesol is used by Candida albicans as a group-sensing molecule for inhibiting filamentation [81]. Researchers have extracted squalene from the grease of rooibos seeds [77,82]. Squalene has the effect of resistance to hypoxia and promoting skin health [82,83] and is mostly used in industrial production and cosmetics [84]. The grease from *S. grosvenorii* seeds has been extracted using a reflux heating method, Soxhlet extraction method, and ultrasonic extraction method, and the grease yield was in the range of 8.05% to 11.46%; the percentage of squalene extracted was close to 12.5% [77].

### 3.6. Other

Mature fruits of *S. grosvenorii* contain 24 inorganic elements, of which 16 are essential trace elements and extensive elements, among which the highest contents are K (12.290 g/kg), Ca (667 mg/kg), and Mg (550 mg/kg) [85,86]. The vitamin C mass fraction in fresh fruit of *S. grosvenorii* can be as high as 313–510 mg/100 g [87]. Researchers carried out an isolation study on the chloroform-extracted fraction of 75% ethanol extract of *S. grosvenorii* and obtained 1-Acetyl-carboline, Cyclo (Pro-Leu), Cyclo (Pro-Ala), maltol, vanillic acid, and β-Sitosterol [88]. Later, magnolol, amber acid [47], and β-Damascenone [89] were discovered.

## 4. Health Benefits

*S. grosvenorii* has a variety of health benefits including metabolic regulation, immunity, respiratory system, antitumor effect (Table 2).

### 4.1. Metabolic Regulation

#### Lower Blood Glucose Aids in the Treatment of Diabetes Mellitus

The metabolic regulating function of *S. grosvenorii* is mainly reflected in the lowering of blood glucose and blood lipid levels and its assistance in the treatment of diabetes. Studies have been undertaken to explore the potential anti-diabetic effects of *S. grosvenorii* [90]. Research has shown that Mogrosides have hypoglycemic and blood-regulating effects. They can reduce the blood sugar by inhibiting the conversion of glucose in food [91]. Studies also have found that Mogroside can alleviate oxidative stress damage in pregnant diabetic rats by activating the Keap1-Nrf2/ARE pathway, thereby protecting the pancreatic tissue. Moreover, Mogroside can improve the glucose and lipid toxicity of pancreatic β-cells and inhibit cell apoptosis. Mogroside V also attenuates gestational diabetes mellitus via the SIRT1 pathway in a rat model [92]. Meanwhile, Mogroside IIIE can inhibit gestational diabetes by activating the adenosine monophosphate-activated protein kinase (AMPK) signaling pathway [93]. Additionally, as a natural plant-based sweetener, Mogroside V can serve as a sugar substitute, offering a high-quality sweetening option for diabetic individuals. Mogroside also has lipid-regulating and weight-reducing functions: research indicates that Mogroside can reduce serum total cholesterol, triglycerides, and low-density lipoprotein levels while increasing high-density lipoprotein levels. It can also inhibit obesity, improve systemic glucose tolerance and insulin sensitivity, induce the browning of white adipose tissue by increasing thermogenic gene expression, and reduce adipose tissue inflammation [94,95].

In addition to Mogroside, other *S. grosvenorii* compounds may also have the potential to reduce blood glucose levels. For instance, researchers have found polysaccharides and polyphenolic compounds in *S. grosvenorii* extracts with hypoglycemic effects. These compounds may act through mechanisms such as promoting insulin secretion, inhibiting intestinal sugar absorption, and enhancing the tissue utilization of glucose [25]. However, there is currently insufficient clinical evidence to confirm the effectiveness of *S. grosvenorii* in diabetes treatment. It must be noted that diabetes is a serious chronic condition that requires comprehensive management strategies, including proper diet, moderate exercise, weight control, medication therapy, and regular monitoring.

### 4.2. Immunity

#### 4.2.1. Antioxidant Effects

The antioxidant properties of *S. grosvenorii* help protect cells from oxidative damage, slow the aging process, enhance immune system function, and reduce the risk of chronic diseases such as cardiovascular diseases, cancer, and *diabetes* [96]. This is because *S. grosvenorii* is rich in antioxidants such as Mogrosides, *S. grosvenorii* polysaccharides, and vitamin C. These components exhibit remarkable antioxidative activity by scavenging free radicals, reducing the occurrence of oxidative reactions, delaying cellular aging, and protecting cells from oxidative damage, thus positively impacting cellular health [97]. Modern pharmacological research on the antioxidative effects of *S. grosvenorii* primarily focuses on the antioxidative effects of Mogrosides and *S. grosvenorii* polysaccharides [8]. Research has found that Mogrosides effectively eliminate free radicals [97]. Both Mogrosides and water extracts of *S. grosvenorii* significantly increase the activities of glutathione peroxidase and superoxide dismutase in high-fat-model mice and reduce the level of malondialdehyde [98]. *S. grosvenorii* extracts can reduce the expression of cytochrome C oxidase 7A2 protein in rat testicular tissue [99]. Additionally, Mogrosides at doses of 0.1 to 10 µg/mL can alleviate oxidative-stress-induced decline in PC12 neuronal cell viability and enhance the antioxidative capacity of PC12 neuronal cells [100]. *S. grosvenorii* polysaccharides *P-1* and *P-2* exhibit excellent scavenging effects on various free radicals, and their scavenging ability increases with the concentration of polysaccharides, with *P-1* demonstrating stronger antioxidative activity than *P-2* [69]. 

#### 4.2.2. Anti-Inflammatory

*S. grosvenorii* and its Mogrosides exhibit significant anti-inflammatory effects by regulating inflammatory pathways, such as inhibiting the release of inflammatory mediators and reducing inflammatory reactions, effectively reducing the severity of inflammation and thereby minimizing tissue damage caused by inflammation [101,102,103]. They help alleviate inflammatory responses and symptoms of inflammation-related diseases. The invasion of the body by Gram-negative bacteria can induce the synthesis of inducible nitric oxide synthase and cyclooxygenase-2 on their cell walls. Mogrosides can inhibit the activation of nuclear factor kappa B (NF-κB) induced by exogenous lipopolysaccharides and blocking the activation of the mitogen-activated protein kinase signaling pathway, thereby reducing the protein levels of inducible nitric oxide synthase and cyclooxygenase-2 [104]. Researchers have found that Mogroside V exerts anti-inflammatory effects by inhibiting the activation, generation, and expression of nuclear factor kappa B, human CCAAT-enhancer-binding protein delta, reactive oxygen species, and activator protein-1/heme oxygenase-1 [105]. Mogroside V significantly inhibited the production of tumor necrosis factor-α (TNF-α), interleukin-1β (IL-1β), interleukin-2 (IL-2), interleukin-6 (IL-6), and nitric oxide (NO), as well as the protein expression of p-P65/P65, cyclooxygenase-2 (COX-2), and inducible nitric oxide synthase (iNOS) in OVA-induced asthmatic mice and LPS-treated RAW 264.7 cells [106]. Additionally, Mogroside IIE has been shown to improve pancreatitis in cell models and mice by downregulating the leukotriene receptor pathway [107].

#### 4.2.3. Antimicrobial Effects

*S. grosvenorii* and its extracts exhibit inhibitory effects on various bacteria and fungi [108]. Researchers have isolated and extracted three compounds, namely hexadecanoic acid, cyclo-(leucine-isoleucine), and sitosterol-3-O-glucoside, from *S. grosvenorii* [79]. Research indicates that all three compounds have inhibitory effects on the biofilm of Escherichia coli. Additionally, *S. grosvenorii* also exhibits inhibitory effects on *Escherichia coli*, *Staphylococcus aureus*, *Bacillus subtilis*, *Rhizopus*, and *Aspergillus* [109]. Although *S. grosvenorii* is considered to have antibacterial properties, the limited number of related studies has resulted in its relatively limited use in antibacterial applications.

### 4.3. Respiratory System

#### 4.3.1. Transformation of Phlegm and Suppression of Coughing

*S. grosvenorii* is widely believed to be efficacious in relieving coughing and reducing phlegm. Therefore, it is traditionally used in Chinese medicine to alleviate symptoms such as coughing and asthma and for moistening the lungs [5]. The antitussive and expectorant functions of *S. grosvenorii* are mainly attributed to its ability to promote relief from inflammation in the throat during coughing, thereby alleviating the discomfort caused by coughing. Studies have shown that water extracts of *S. grosvenorii* and Mogroside V can significantly reduce the amount of coughing in mice and prolong the cough latency period [110]. Mogroside V can also significantly increase the volume of phenol red excreted by mice and antagonize histamine-induced ileal contraction and tracheal spasming [111]. Researchers have found that the water extract of *S. grosvenorii* can improve pharyngitis and significantly inhibit the expression of interleukin-1β, -6, and tumor necrosis factor-α in the serum of model animals [112]. Mogroside V can alleviate ovalbumin-induced airway inflammation, specifically by reducing airway hyperreactivity in asthmatic mice, thus decreasing the levels of interleukin-4, -5, -13, and serum ovalbumin-specific immunoglobulin E and G1 [113]. Additionally, *S. grosvenorii* contains other chemical components such as flavonoids and polysaccharides. According to current research, flavonoids may help to alleviate inflammation and relieve coughing and throat discomfort, thereby exerting certain respiratory system health benefits [114,115]. Polysaccharides may have immune-regulating and antiviral effects, which can help resist respiratory infection and alleviate coughing to some extent [116,117].

#### 4.3.2. Prevention of Pulmonary Fibrosis

*S. grosvenorii* extracts, especially Mogroside, have been shown to be very useful in preventing pulmonary fibrosis [118,119]. Mogroside IIIE can effectively prevent pulmonary fibrosis by modulating the Toll-like receptor 4/myeloid differentiation factor-88/nuclear factor kappa B signaling pathway, thereby inhibiting pulmonary inflammation and extracellular matrix deposition [120]. Furthermore, research indicates that Mogroside IVE possesses the ability to alleviate liver fibrosis. Its mechanism of action may involve inhibiting the Toll-like receptor 4 signaling pathway and hypoxia-inducible factor-1α [121].

### 4.4. Antitumor Effect

According to current experimental research, Mogrosides have demonstrated some antitumor functions [122,123]. *S. grosvenorii* alcohol also significantly inhibits the proliferation of prostate cancer DU145 cells, liver cancer HepG2 cells, lung cancer A549 cells, and nasopharyngeal carcinoma CNE1 and CNE2 cells [124]. The inhibitory effect on CNE1 cell proliferation is significant and dose dependent. It may be achieved by promoting pro-apoptotic genes such as caspase-3 and Bax proteins and inhibiting anti-apoptotic genes such as lymphoma/leukemia-2 and Survivin B in tumor cells [122,123]. Mogrosides exhibited anticancer activity against PC-3 and T24 cells, significantly reducing cell viability and ultimately inducing apoptosis [125]. In addition, enrichment analysis with molecular docking indicated that Mogrosides V may be a potential therapeutic agent for the alleviation of COVID-19 in ovarian cancer patients [126]. However, most of the current research on the anti-cancer effects of *S. grosvenorii* is still in the laboratory and animal stages, and there is not enough clinical evidence to support its effectiveness in the human body.

### 4.5. Other Effects

#### 4.5.1. Mental System Regulation

*S. grosvenorii* possesses the ability to regulate the nervous system. Studies have shown that Mogroside V and its metabolite, 11-oxo-mogroside V, can inhibit neuron damage induced by dextromethorphan malate by promoting neurite outgrowth and inhibiting cell apoptosis [127]. Furthermore, Mogroside V can alleviate rot-induced neurotoxicity in a PD model [128].

#### 4.5.2. Fatigue Reduction

*S. grosvenorii* also exhibits a fatigue-relieving effect. Research indicates that extracts of *S. grosvenorii* can alleviate fatigue by significantly increasing hepatic glycogen and muscle glycogen concentrations in fatigued mice [129]. Additionally, it increases testosterone levels in rats after weight-loaded swimming training, improves substance metabolism, and significantly enhances rats’ anti-fatigue capacity [130]. Furthermore, studies suggest that Mogroside V can reduce intracellular erythropoietin levels, enhance mitochondrial function, and promote the development of porcine oocytes in vitro [131].

#### 4.5.3. Hepatoprotective Effects

The hepatoprotective effects of *S. grosvenorii* primarily manifest in detoxification, anti-inflammatory, antioxidant, and anti-fibrotic activity, and the promotion of liver cell regeneration. These effects contribute to the maintenance of normal liver function and the reduction in the risk of liver diseases. Regarding the hepatoprotective effects of Mogroside, modern pharmacology mainly focuses on its anti-liver fibrosis properties. Studies have shown that Mogroside may exert anti-liver fibrosis effects by inhibiting the expression of transforming growth factor-β1 and type I collagen protein and mRNA, thereby suppressing hepatic stellate cell activation and hepatocyte apoptosis, inhibiting collagen synthesis, and promoting extracellular matrix degradation [132,133]. Additionally, research has demonstrated that Mogrosides have a protective effect on acute liver injury induced by CCl4 in mice and preventive and therapeutic effects on chronic liver injury induced by CCl4 in rats [121]. Furthermore, water extracts of *S. grosvenorii* can significantly improve the intestinal microbiota of mice with non-alcoholic fatty liver disease [134].

#### 4.5.4. Constipation

*S. grosvenorii*, traditionally known for its efficacy in moistening the intestines and relieving constipation, exhibits effects on the digestive system. Research has found that water extracts of *S. grosvenorii* can enhance spontaneous activity in isolated intestines of rabbits and dogs and exert an antagonistic effect on intestinal contraction and relaxation [135]. Furthermore, *S. grosvenorii* water extracts have a laxative effect on both normal and constipated mice and exhibit antispasmodic effects on isolated intestines [136,137]. Mogroside increases the frequency, quality, and ink propulsion rate of mouse defecation, thereby expediting bowel movements [11]. These findings suggest that extracts of *S. grosvenorii* can improve the digestive system, primarily by alleviating constipation.

**Table 2:** Pharmacological activities and health benefits of *S. grosvenorii*.

NO.	Ingredients	Pharmacological Activities	Health Benefits	Ref.
1	Mogroside	Hypoglycemic and blood-regulating effects	Aids in the treatment of diabetes mellitus	[91]
2	Mogroside (Mogroside V)	Reducing oxidative stress damage	Reducing gestational diabetes	[92,93]
3	Mogroside	Functions of regulating blood lipids and reducing body weight	Aids in the treatment of obesity	[94,95]
4	*S. grosvenorii* extracts	Enhancing the tissue utilization of glucose	Aids in the treatment of obesity	[25]
5	Mogrosides and water extracts of *S. grosvenorii*	Increased glutathione peroxidase and superoxide dismutase (sod) activity, and lowering malondialdehyde level	Antioxidation	[98]
6	*S. grosvenorii* extracts	Reduces the expression of cytochrome C oxidase 7A2 protein	Antioxidation	[99]
7	Mogroside	Alleviates an oxidative-stress-induced decline in PC12 neuronal cell viability	Enhances the antioxidative capacity of PC12 neuronal cells	[100]
8	*S. grosvenorii* polysaccharides P-1 and P-2	Excellent scavenging effects on various free radicals	Antioxidation	[69]
9	Mogroside	Protein levels of inducible nitric oxide synthase and cyclooxygenase-2	Minimizing tissue damage caused by inflammation	[104]
10	Mogroside V	Inhibits inflammation protein expression	Exerts anti-inflammatory effects	[105,106]
11	Mogroside IIE	Regulating pathway	Treatment of acute pancreatitis	[107]
12	*S. grosvenorii* extracts	Can inhibit bacteria and fungi	Antibacterial	[108,109]
13	*S. grosvenorii* and Mogroside V	Reduces a cough	Treating a cough	[110]
14	Mogroside V	Eases tracheal spasms	Treating a cough	[111,113]
15	Water extracts of *S. grosvenorii*	Improves the swallow	Treating a cough	[112]
16	Flavonoids in *S. grosvenorii*	Alleviates inflammation and relieves a cough and throat discomfort	Treatment of the respiratory system	[114,115]
17	Polysaccharides in *S. grosvenorii*	Immune regulation and antiviral effect	Works against respiratory infections and relieves a cough	[116,117]
18	*S. grosvenorii* extracts (especially Mogroside)	Relieves pulmonary fibrosis	Prevention of pulmonary fibrosis	[118,119]
19	Mogroside IIIE	Inhibition of pulmonary inflammation and extracellular matrix deposition	Prevention of pulmonary fibrosis	[120]
20	Mogroside IVE	Inhibiting the Toll-like receptor 4 signaling pathway and hypoxia-inducible factor-1α	Prevention of pulmonary fibrosis	[121]
21	Mogrosides	Antitumor	Adjuvant treatment of cancer	[122,123,125,126]
22	*S. grosvenorii* alcohol	Inhibition of cancer cells	Adjuvant treatment of cancer	[124]
23	Mogroside V and its metabolite, 11-oxo-mogroside V	Inhibition of neuronal injury	Neuroprotection	[127,128]
24	*S. grosvenorii* extracts	Concentrations of liver glycogen and muscle glycogen and the level of testosterone were increased	Alleviate fatigue	[129,130]
25	Mogrosides	Anti-liver fibrosis and protection of liver injury	Hepatoprotective effects	[121,132,133]
26	*S. grosvenorii* extracts and Mogrosides	Enhanced bowel movement	Ease constipation	[11,135,136,137]

## 5. Application in Medicine, Nutrition, and Food

### 5.1. Application in Medicine

**Figure 5:**
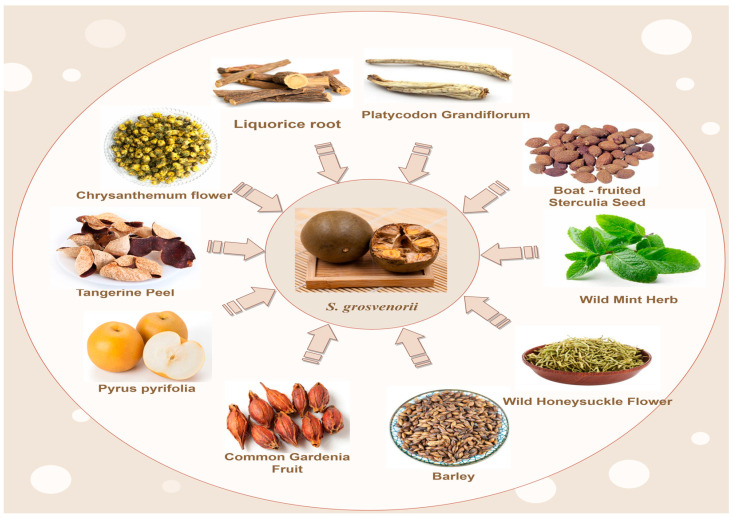
*S. grosvenorii* and other herbs in traditional Chinese medicine for medicinal tea applications.

As a traditional medicinal plant, *S. grosvenorii* has a long history of use in China. It has high nutritional value and was one of the first precious Chinese herbal medicines to be listed by the Chinese government in the homology of medicine and food [3]. In Traditional Chinese Medicine (TCM) theory, *S. grosvenorii* is cool in nature, sweet in taste, belongs to the lung and large intestine meridian, has the effects of clearing away heat and moistening the lungs, facilitating the pharynx and opening up the voice, and moistening the intestines and laxatives, and can be used for the treatment of lung-heat and a dry cough, a sore throat and loss of voice, and intestinal dryness and constipation [138]. The fruit of *S. grosvenorii* is usually harvested in autumn, when the color of the fruit changes from lighter green to dark green, and after a few days it is dried at a low temperature. The dosage is 9–15 g [15]. Chinese people often like to use *S. grosvenorii* and other herbs, such as tangerine peel, chrysanthemum flower, licorice root, *Platycodon grandiflorus*, boat-fruited Sterculia seed, wild mint herb, wild honeysuckle flower, barley, common gardenia fruit, *Pyrus pyrifolia*, etc. The herbs are used to make tea and treat dry and hoarse throats caused by staying up late and other reasons [138] (Figure 5). Another application in traditional Chinese medicine is *S. grosvenorii* broth, where *S. grosvenorii* is stewed with pork to treat physical weakness after a prolonged cough. A recipe from the “Ling Nan Cai Yao Lu” describes *S. grosvenorii* pork soup: take 30–60 g of *S. grosvenorii* and 100 g of lean pork, then add water to decoct. This treatment is used for throat diseases such as lung deficiency or a consumptive cough [138].

### 5.2. Application in Nutrition

*S. grosvenorii* is a healthy fruit choice, low in sugar and calories and rich in antioxidants and nutrients. The sweet taste of *S. grosvenorii* is mainly due to natural Mogroside V rather than glucose or other carbohydrates. The calorie content of *S. grosvenorii* is very low, with only about 25 calories per 100 g. This makes it a healthy snack choice for weight control [139]. It has a very low effect on blood glucose levels and is suitable for people with diabetes or those pursuing a low-sugar diet [140]. This allows it to be used as a sweetener or snack or to be added to beverages and foods to provide natural sweetness and nutritional value [141]. Mogroside V and Siamenoside II are the major metabolites of the fruit’s pulp and comprise more than 30% of the total sweetener compounds [19,140]. *S. grosvenorii* is also approved as a medicine food homology (MFH) plant due to its nutritional and (various) pharmaceutical properties [142]. To date, more than 60 Mogrosides have been identified in *S. grosvenorii*, of which the major compounds are cucurbitane triterpene glycosides such as Mogroside (IIE, III, IIIE, IVA, IV, V), Iso-Mogroside V, 11-Oxomogroside (IA, IIE, V), Siamenoside (I and II), etc. These molecules have displayed a marked variation in sweetness intensity, taste profile, and medicinal usage [5,143]. *S. grosvenorii* is rich in vitamin C (400 mg to 500 mg per 100 g of fresh fruit) as well as glycosides, fructose, glucose, proteins, and lipids. These nutrients are essential for maintaining good health and supporting the function of the immune system. *S. grosvenorii* contains soluble dietary fiber, which not only helps to promote the health of the digestive system and maintain the normal function of the intestine but also helps to control blood sugar and cholesterol levels. *S. grosvenorii* has the advantages of being low in sugar, low in calories, and high in dietary fiber, which makes it suitable for healthy eating and controlling the calorie intake. Compared to other fruits, *S. grosvenorii* derives its sweetness from its Mogroside content rather than being high in sugar, which makes it a deliciously sweet option while containing few calories for people concerned about their calorie intake.

### 5.3. Application in Food

*S. grosvenorii* is popular in food. It has a distinctive and very sweet flavor that makes it an ideal ingredient in a wide variety of foods and beverages, and extracts of *S. grosvenorii* are often used as a natural sugar substitute in the preparation of juices, beverages, jellies, candies, pastries, and other delicacies, adding a special sweetness to these foods. As *S. grosvenorii* contains a wide range of nutrients and chemicals that are beneficial to the health, people are also more favorably disposed towards the foods in which it is used, making rooibos popular in the food industry [6,19,144]. *S. grosvenorii* is widely used in tea drinks; due to the challenges in preserving fresh *S. grosvenorii*, the majority of them are sold in the market through sun-drying or freeze-drying [19,144]. There is also a wide range of applications for *S. grosvenorii* and its extracts in other beverages, such as juicing *S. grosvenorii*, drinking the pulp or extracts alone, or making blended juices with other fruits. *S. grosvenorii* can add a natural sweet and sour taste and fruity flavor to beverages. Mogroside in *S. grosvenorii* can be added as a natural sweetener to beverages such as milk and fruit teas [19,144]. Sugar-free beverages have also become popular in recent years with those who like to drink beverages but are afraid of consuming too much sugar as a result of this [6]. In particular, NAIXUE, a well-known tea beverage company in China, began to replace synthetic sugar substitutes such as sucralose with *S. grosvenorii* sugar substitute extract in 2020. Sugar production company Taikoo Sugar also uses *S. grosvenorii* sugar substitutes made from *S. grosvenorii* extract. In addition, *S. grosvenorii* and its extracts are also used as an additive in pastries, bread, or ice cream to add flavor. Moreover, *S. grosvenorii* is often used in throat lozenges and throat tea products, which are widely sought after in the market. Modern Chinese medicine product companies such as Tong Ren Tang, Nien Ci An, and Hong Fu Tang also sell relevant products [144]. 

## 6. Toxicology

Relatively few studies have been conducted on the toxicological aspects of *S. grosvenorii*, and no large-scale toxicological studies have been reported. However, some studies have provided a preliminary assessment of the safety of *S. grosvenorii*, particularly the toxicological potential of *S. grosvenorii* extract or its constituents. Researchers found no significant changes in general appearance, body weight, food and water consumption, hematological and serum biochemical parameters, organ weights, and histopathological findings between the control and treated groups in a study in which mice were fed Siraitia grosvenori extract for 13 weeks [145]. Gavage studies were carried out on male and female dogs using *S. grosvenorii* extract and no significant toxicological effects were discerned from the observational records, including clinical observations, body weight, food consumption, hematology, blood chemistry, urinalysis, gross necropsy, organ weights, and histopathology results [146]. A report on *S. grosvenorii* extract by the EFSA Panel on Food Additives and Flavourings (FAF) suggests that a chronic dietary intake of high doses of *S. grosvenorii* extract in mice may lead to a number of undesirable effects such as abnormalities in liver and kidney function and reduced testicular weight in male mice [147]. However, these findings require further validation and in-depth study. In addition, biological effects of other chemical components and interactions of Luo Han Guo glycosides with other compounds cannot yet be ruled out.

## 7. Conclusions and Future Perspectives

Research on *S. grosvenorii* and Mogroside is still relatively limited. The available laboratory and animal studies indicate that Mogroside is a natural sweetener with excellent pharmacological effects, including anti-inflammatory, antioxidant, hypoglycemic, lipid-regulating, and anti-diabetic properties. Consequently, its use is gradually becoming more widespread. Future research should focus on conducting more clinical studies to investigate the exact efficacy and safety of *S. grosvenorii* and Mogroside. These studies could involve human trials of *S. grosvenorii* extracts or related compounds, to assess their efficacy, dosage, and administration in treating inflammation, oxidative stress, diabetes, and other diseases. Additionally, further studies are needed to understand the interactions between *S. grosvenorii* and other drugs or treatments, as well as potential adverse effects. Overall, Mogroside is of interest as a natural anti-inflammatory and cytoprotective agent and as a sweetener capable of regulating blood glucose and lipids. However, further scientific research is necessary to support its effectiveness and safety in various applications.

## Data Availability

No new data were created or analyzed in this study.
